# Pre- and post-weaning diet alters the faecal metagenome in the cat with differences vitamin and carbohydrate metabolism gene abundances

**DOI:** 10.1038/srep34668

**Published:** 2016-11-23

**Authors:** Wayne Young, Christina D. Moon, David G. Thomas, Nick J. Cave, Emma N. Bermingham

**Affiliations:** 1 Food Nutrition & Health Team, Food & Bio-based Products Group, AgResearch Grasslands, Palmerston North, New Zealand; 2 Rumen Microbiology Team, Animal Science Group, AgResearch Grasslands, Palmerston North, New Zealand; 3 Centre of Feline Nutrition, Institute of Veterinary, Animal and Biomedical Sciences, Massey University, Palmerston North, New Zealand

## Abstract

Dietary format, and its role in pet nutrition, is of interest to pet food manufacturers and pet owners alike. The aim of the present study was to investigate the effects of pre- and post-weaning diets (kibbled or canned) on the composition and function of faecal microbiota in the domestic cat by shotgun metagenomic sequencing and gene taxonomic and functional assignment using MG-RAST. Post-weaning diet had a dramatic effect on community composition; 147 of the 195 bacterial species identified had significantly different mean relative abundances between kittens fed kibbled and canned diets. The kittens fed kibbled diets had relatively higher abundances of *Lactobacillus* (>100-fold)*, Bifidobacterium* (>100-fold), and *Collinsella* (>9-fold) than kittens fed canned diets. There were relatively few differences in the predicted microbiome functions associated with the pre-weaning diet. Post-weaning diet affected the abundance of functional gene groups. Genes involved in vitamin biosynthesis, metabolism, and transport, were significantly enriched in the metagenomes of kittens fed the canned diet. The impact of post-weaning diet on the metagenome in terms of vitamin biosynthesis functions suggests that modulation of the microbiome function through diet may be an important avenue for improving the nutrition of companion animals.

There is increasing interest in the effects of diet on the composition of intestinal microbiota in domesticated cats and dogs due to the clear links between microbiota and pet health^1^,^2^,^3^,^4^. Complementary to the changes in microbial community composition associated with diet are the effects of diet on the function of the intestinal microbiome. Investigating these in parallel gives insight not only into the effect of diet on the taxonomic composition of the microbiome, but into the impact that this has on potential microbiome function.

While much research has investigated the effects of specific dietary components (e.g., protein, carbohydrate, or fibre) on intestinal microbial composition in laboratory settings, pets in the home environment are typically fed specific formats of pet food, generally either kibbled or canned diets. There is relatively little research on the impact of dietary format, but its role in pet health is of great interest to pet food manufacturers and pet owners alike. Commonly six dietary components are discussed in terms of dietary format – carbohydrate, protein, fat, water and vitamin and mineral contents. Kibbled diets tend to have higher carbohydrate contents and lower protein levels, whereas canned diets have very low carbohydrate content, and medium to high levels of protein and fat. Canned diets also contain approximately 75–80% moisture. Our previous research has shown large differences in the composition of faecal microbiota in the domestic cat associated with either short-term changes in diet^5^ or following the feeding of kibbled and canned post-weaning diets^6^.

Prior research has shown that taurine status of cats is affected by dietary format, with taurine availability lower in canned diets compared to kibbled diets. Intestinal microbiota have a role in overall taurine status^7^,^8^. Additionally, it has been reported in recent reviews that Maillard reaction products (chemical reactions between amino acids and sugars) are high in commercially available pet foods, especially canned formulations^9^ and that they have the potential to impact on the health of the pet, although the mechanisms by which this may occur are not clear^10^.

Although vitamins can be obtained from a variety of foods, some vitamins (e.g., vitamin B1 (thiamine), vitamin B9 (folic acid) and vitamin B12 (cobalamin)) can also be provided through microbial *de novo* biosynthesis^11^,^12^. Indeed, microbe-derived vitamins may be of particular importance when diets are deficient in these vitamins. Thiamine deficiency has been reported in cats and has largely been attributed to heat treatment during the canning process^13^,^14^. While it has been suggested that kibbled diets may increase thiamine requirements due to their high carbohydrate content^14^, recent literature suggests that thiamine concentrations in canned food may be below the recommended amounts for adult cats^15^.

To our knowledge, four studies have examined the function of the intestinal microbiota in the domestic cat^16^,^17^,^18^,^19^, of which two investigated dietary parameters (fibre^17^ and protein^18^ levels) in weaned kittens, but the impacts of pre-weaning diet on the function of intestinal microbiota in relation to vitamin synthesis have not yet been investigated. Furthermore, the majority of cats in home settings are fed either canned or kibbled diets, yet few published studies have examined the impacts of these diet formats on the composition and function of intestinal microbiota in domestic cats. Our previous research using sequencing of 16S rRNA gene amplicons^6^ showed that pre-weaning consumption of a canned versus a kibbled diet altered the faecal relative abundance of several taxa, including *Solobacterium*, *Peptococcaceae*, *Clostridium,* and *Megamonas*.

The aim of the present study was to investigate the effects of pre-weaning (gestation and lactation) diet and post-weaning diet on the composition and function of faecal microbiota in the domestic cat through metagenome shotgun sequencing, focussing on vitamin and taurine metabolism.

## Results and Discussion

### Apparent digestibility

The growth rate of the kittens has been published previously^6^, however, kittens fed the canned diet were heavier (*P* < 0.05) by 17 weeks of age than those fed the kibbled diets (1.3 vs 1.4 kg (SEM 0.1) kg). The apparent digestibility of energy and protein was altered (*P* < 0.05) in response to post-weaning diet (Table 1), while fat digestibility (*P* = 0.063) was similar between diets. Faeces from cats fed the canned diet contained significantly more energy (*P* = 0.006) and fat (*P* = 0.012), while faecal protein content was similar between cats on the two diets (*P* = 0.503).

### Community taxonomic composition

Paired-end sequences were deposited in the MG-RAST database and are publically accessible with the following metagenome identifiers; 4629274.3, 4629275.3, 4629276.3, 4629277.3, 4629278.3, 4629279.3, 4629280.3, 4629281.3, 4629282.3, 4629283.3, 4629284.3, 4629285.3, 4629286.3, 4629287.3, 4629288.3, 4629289.3, 4629290.3, 4629291.3, 4629292.3, and 4629293.3.

Alignment against the M5NR database resulted in an average of 3,663,452 paired end sequences per sample (minimum 1,734,438, maximum 6,577,103, standard deviation 1,163,541) that could be taxonomically classified.

**Figure 1:**
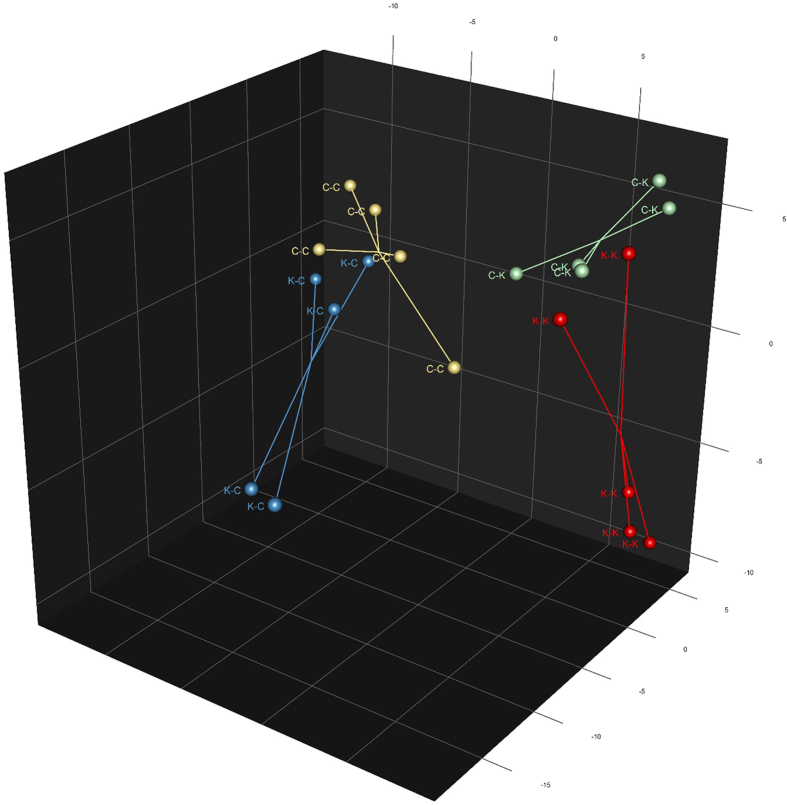
Scores plot from PCA of kittens’ faecal microbiota composition at the species level. The first letter indicates the mother’s diet during pregnancy and lactation and the second letter indicates the kitten’s diet; canned (C) or kibbled (K). Lines converge at the spatial centroid for each treatment group. Diet K-Diet K (K-K) n = 3 females, n = 2 males. Diet C-Diet K (C-K) n = 3 females, n = 2 males. Diet K-Diet C (K-C) n = 3 females, n = 2 males. Diet C-Diet C (C-C) n = 4 females, n = 1 male.

Consistent with our previous study^6^, the post-weaning diet had a considerable effect on the composition of faecal microbiota (Fig. 1). Of the 195 taxa identified at the genus or species level, 147 had significantly different mean relative abundances (FDR < 0.05) between kittens fed kibbled and canned diets (i.e. C-K and K-K kittens compared to K-C and C-C kittens; Supplementary Table S1–S5xlsx). Overall, the kittens fed kibbled diets post-weaning had substantially higher relative abundances of bacteria from the *Lactobacillus*, *Bifidobacterium*, and *Collinsella* genera compared to kittens fed the canned diet post-weaning (Table 2). In our previous study using 16S amplicon sequencing, an increase in *Lactobacillus* of similar magnitude was also observed in kittens fed the kibbed diet^6^. However, *Bifidobacterium* was not detected in our previous study and the proportions of *Collinsella* were <1%^6^, whereas in our current study, *Collinsella* made up >10% of the microbiota in kittens fed the kibbled diet. This discrepancy may be explained by the differences in sequencing methods used; reliable detection of *Bifidobacterium* using V1-V3 primers, such as those used in our previous study, can be problematic^20^, and PCR amplification biases are avoided by shotgun sequencing methods.

In general, *Lactobacillus* and *Bifidobacterium* are recognised as adept carbohydrate utilising bacteria^21^,^22^, with genomes containing a high number genes encoding a wide range of carbohydrate transport and utilisation functions^21^,^23^,^24^, so their enrichment in the community of kittens fed the kibbled diets is consistent with complex carbohydrate diet studies in other mammalian species^25^,^26^,^27^,^28^. In contrast, kittens fed the canned diet post-weaning had higher relative abundances of bacteria from the *Fusobacterium*, *Bacteroides* and *Clostridium* genera (Table 2), all of which include representatives that have proteolytic activity^29^,^30^,^31^. *Fusobacterium* are commonly found in the faeces of healthy dogs^32^,^33^. Furthermore, because *Fusobacterium* are proteolytic bacteria^34^ they have been associated with high protein diets in kittens^35^, adult cats^5^,^36^ and dogs^37^. *Bacteroides* are known to utilise host mucin glycans^38^,^39^ in the absence of dietary carbohydrates, which may also partially explain the success of these bacteria in kittens fed the canned diet. Sequences aligning with sialidase-1, Fucose 4-O-acetylase, and N-acetylneuraminate lyase, which are involved in mucin degradation^40^, were between 7 and 80-fold higher (FDR <0.05; Supplementary Table S6.xlsx), in kittens fed the low carbohydrate, canned diet. This is consistent with greater utilisation of host mucins, and may therefore partially explain the success of bacteria such as *Bacteroides* in kittens fed the canned diet. However, the overall relative abundance of these sequences was low, comprising <1% of the overall metagenome.

In addition to effects of post-weaning diet, the type of diet consumed by the dams during pregnancy and lactation had a measurable impact on the kitten’s microbial community 17 weeks post-weaning (Fig. 1). *Streptococcus* spp. were particularly prominent in kittens of mothers fed the kibbled diet that were also fed the kibbled diet (K-K kittens; Table 2); these species collectively made up over 46% of the communities, on average, in these kittens. Of these, *S. infantarius* and *S. gallolyticus* were dominant, making up 15.2% and 14.3% of the community in K-K kittens, respectively (Supplementary Table S1xlsx). In contrast, streptococci were only a minor component of the microbiota (<1%) in kittens of mothers fed the canned diet, which had been weaned onto the kibbled diet (C-K). Streptococci were also in low abundance in K-C and C-C kittens (0.13% and 0.09%, respectively). Streptococci are a diverse group of microbes, and are efficient fermenters of simple sugars. They are among the earliest colonisers of the gastrointestinal tract, where they may be detected within a day of birth^41^. The high relative abundance of streptococci observed in kittens for which kibbled diets were fed both pre- and post-weaning likely reflects the maternal colonisation source in establishing this taxon, and the continued selection for it by the kibbled diet. Genus level data from our previous study^6^ indicated that the mothers of these kittens also had higher levels of streptococci than mothers of kittens with low levels of streptococci. While streptococci were of low relative abundance in the C-K group compared to the K-K group, lactobacilli were 2-fold higher. Therefore, it is possible that the lactobacilli, along with other bacteria, were filling the carbohydrate utilising niche that would otherwise be occupied by the streptococci.

Other taxa that have relative abundances affected by both the mother’s diet and the weaning diet included *Escherichia* and *Shigella*, which consisted of 3.2% and 4.5% of the community from K-C kittens, respectively, but formed <1% of the community in all other groups (Table 2; Supplementary Table S2xlsx). These results show that early microbiome seeding events can influence how the microbiome responds to dietary changes later in life. In particular, the K-C group exhibited a significant relative increase in numerous members of the *Gammaproteobacteria*, such as *Escherichia coli*, *Shigella* spp., *Salmonella enterica*, *Citrobacter* spp., and *Klebsiella* spp. (Supplementary Table S1xlsx). Collectively, the *Gammaproteobacteria* made up 8.1% of the community in K-C kittens, but were not more than 0.3% of the community in K-K, C-K, or C-C kittens (Supplementary Table S4xlsx). The reasons for the prevalence of *Gammaproteobacteria* within the K-C treatment are unclear.

### Community metagenome function

Hierarchical functional analysis of paired end sequences with a minimum identity of 80% resulted in an average of 909,272 paired end sequences per sample (minimum 95,962, maximum 2,621,630, standard deviation 705,180) that could be functionally classified.

**Figure 2:**
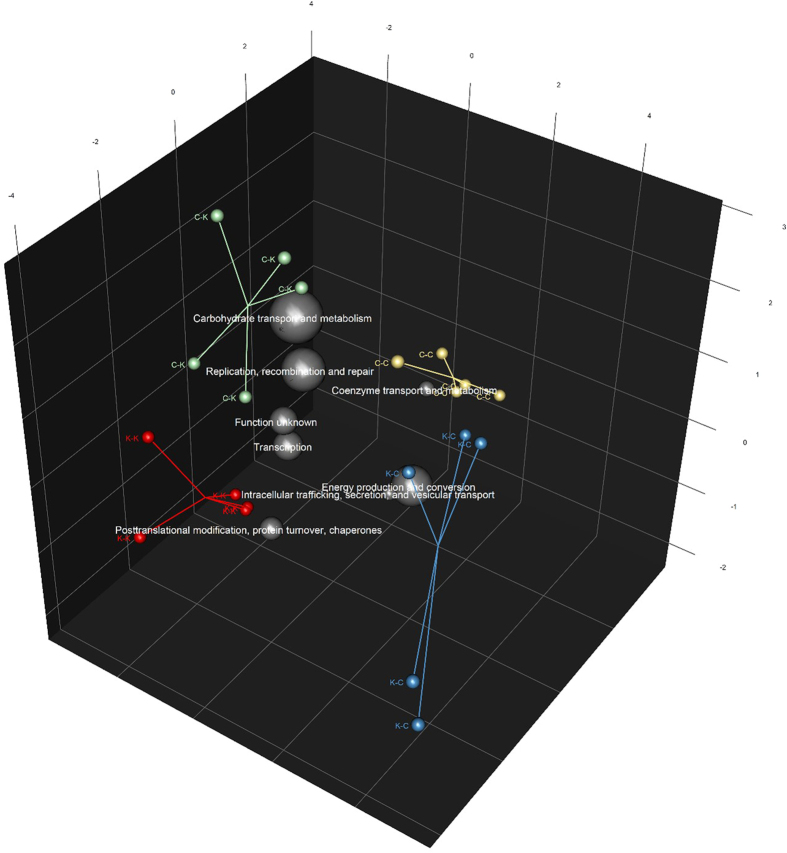
Scores biplot from partial least squares discriminant analysis (PLS-DA) of kittens’ faecal metagenome COG-predicted functional classifications. The first letter indicates the mother’s diet during pregnancy and lactation and the second letter indicates the kitten’s diet; canned (C) or kibbled (K). Lines converge at the spatial centroid for each treatment group. Grey spheres show the COG functions that best discriminate the groups from each other. The size of the spheres are proportional to the relative abundance of that COG function. The spatial position of each COG function is plotted as a weighted average of the coordinates of all samples. Diet K-Diet K (K-K) n = 3 females, n = 2 males. Diet C-Diet K (C-K) n = 3 females, n = 2 males. Diet K-Diet C (K-C) n = 3 females, n = 2 males. Diet C-Diet C (C-C) n = 4 females, n = 1 male.

**Figure 3:**
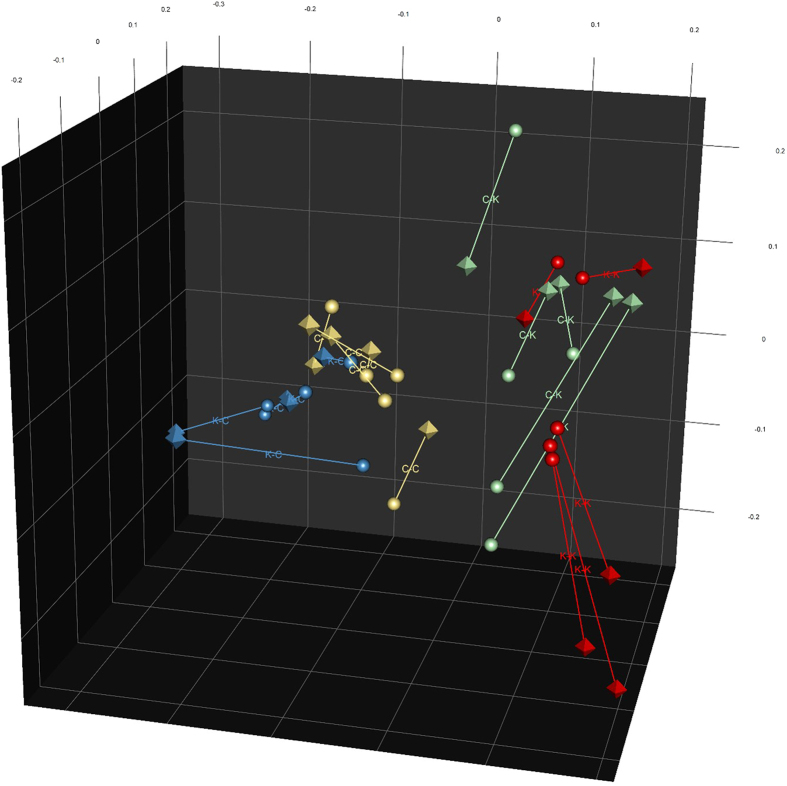
Scores plot of Procrustes rotation analysis of the kittens’ faecal microbial taxonomic composition and metagenome COG-predicted functions profiles. Octahedrons show taxonomic projection coordinates and spheres show COG coordinates for each sample. Lines join taxonomic and COG profiles from the same sample, with closer proximity indicating greater similarity between the two projections. The first letter indicates the mother’s diet during pregnancy and lactation and the second letter indicates the kitten’s diet; canned (C) or kibbled (K). Diet K-Diet K (K-K) n = 3 females, n = 2 males. Diet C-Diet K (C-K) n = 3 females, n = 2 males. Diet K-Diet C (K-C) n = 3 females, n = 2 males. Diet C-Diet C (C-C) n = 4 females, n = 1 male.

While the maternal diet during pregnancy and lactation had some observable effects on the microbial community composition in the kittens at 17 weeks of age, they had less impact on the predicted metagenome function composition (Supplementary Table S6xlsx). Comparisons of the kitten’s faecal metagenomes, classified using the Clusters of Orthologous Groups (COG) of proteins database and analysed using permutation MANOVA, showed a significant effect of post-weaning diet (*P* = 0.001). Although no effect from the maternal diet was observed (*P* = 0.687), there was a significant interaction between maternal diet and post-weaning diet (*P* = 0.031). This observation was supported by discriminant analyses which showed that the kitten’s faecal metagenomes could be differentiated based on the mother’s diet (Fig. 2). Comparisons of the metagenome gene functions with community taxonomic compositions via Procrustes rotation analysis also showed good agreement between the two analyses (correlation = 0.76, *P* = 0.001; Fig. 3). The lesser differences observed on the composition of the metagenome compared to the microbial taxonomic composition is a well reported phenomenon^42^, suggesting that a high degree of genetic redundancy exists in the microbial community. Nevertheless, in our study, the post-weaning diet clearly impacted the kitten’s metagenome composition.

### Carbohydrate and energy metabolism

The differences observed in faecal microbial communities, and faecal protein and energy content, appear to be reflected in some of the metagenome functions observed. The higher relative abundance of ostensibly adept carbohydrate utilising bacteria in kittens fed the kibbled diet was associated with a higher proportion of sequences that mapped to the COG category “Carbohydrate transport and metabolism” (Table 3; FDR = 0.017). The higher energy content in the faecal material of kittens fed the kibbled diet was also reflected in a greater proportion of sequences that mapped to the COG category “Replication, recombination and repair”, indicating greater bacterial proliferation in those kittens (Table 3; FDR = 0.006). Conversely, the lower energy content in faecal material from kittens fed the canned diet was associated with increased relative abundance of sequences that mapped to the COG category “Energy production and conversion” (Table 3; FDR = 0.006).

Associated with the apparent difference in energy metabolism, kittens from the K-C group had a significantly higher number of sequences with hits to genes encoding phosphogluconate dehydratase [EC:4.2.1.12] compared to the other treatment groups (interaction FDR = 0.02; Supplementary Table S6xlsx). This enzyme is a key component of the Entner-Doudoroff (ED) pathway^43^, which is an alternate mechanism to the more commonly used Embden-Meyerhof-Parnas (EMP) pathway for generating ATP. The ability to use the ED pathway is a characteristic feature of many *Gammaproteobacteria*^44^,^45^,^46^, which were also the most relatively abundant in the K-C kittens. Although less efficient than the EMP pathway in terms of ATP generated per molecule of glucose, the ED pathway requires less enzyme machinery and therefore has lower protein requirements^46^,^47^. The lower protein content in faeces from kittens fed the canned diet may be a contributing factor in the increased relative abundance of *Gammaproteobacteria* and use of the ED pathway in the K-C kittens.

Recently, Deusch *et al*.^18^ reported that post-weaning changes in the protein: carbohydrate ratio (high protein/low carbohydrate (HP/LC) and medium protein/medium carbohydrate (MP/MC)) changed the structure and function of the faecal microbiome of the cat. The top 5 KEGG functions were replication and repair (approx. 12% of sequences), amino acid metabolism (approx. 12% of sequences), carbohydrate metabolism (approx. 11% of sequences), translation (approx. 8% of sequences), nucleotide metabolism (approx. 7% of sequences)^18^. Our findings show good agreement with these results.

### Vitamin biosynthesis and metabolism

A number of vitamins are metabolised within the intestinal tract of mammals^48^. These include vitamin K and the vitamin B family. The vitamin B family encompasses a range of vitamins including vitamin B1 (thiamine), B2 (riboflavin), B3 (niacin), B6 (pyridoxine), B9 (folic acid) and B12 (cobalamin). In comparison to other carnivores (e.g., dogs), cats appear to have a higher requirement for several B-vitamins that arise from microbial synthesis such as thiamine, riboflavin, niacin, pyridoxine and folic acid^49^,^50^. Therefore, it was of interest to understand the effects of diet format on pathways involved in vitamin B metabolism in the metagenome of the cat.

Thiamine deficiency has been reported in cats fed canned diets and has largely been attributed to heat treatment during the canning process^13^,^14^, preservatives such as sulphur dioxide^51^,^52^ or fish-based diets^53^. While it has been suggested that kibbled diets may increase thiamine requirements due to their high carbohydrate content^14^, recent literature suggests that thiamine concentrations in canned food may be below the recommended amounts for adult cats^15^. As for all mammals, thiamine cannot be synthesised by the cat, instead it relies on microbial *de novo* biosynthesis^54^ or ‘salvaging’ pathways^54^,^55^,^56^.

**Figure 4:**
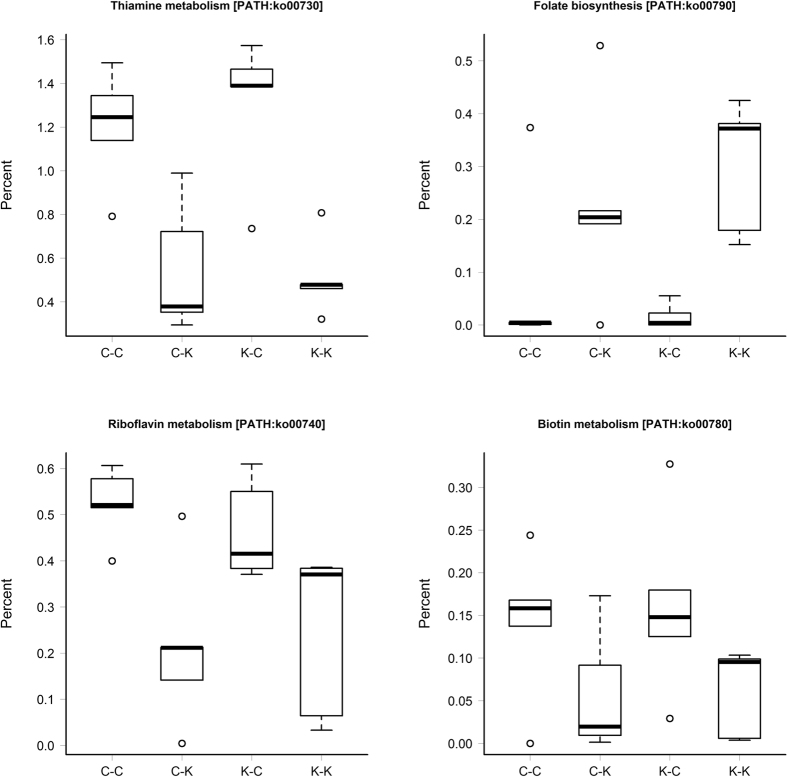
Vitamin B related KO pathways that showed significantly different relative abundances (FDR < 0.05) between kittens fed the canned (C) diet (C-C and K-C) or the kibbled (K) diet (C-K and K-K). Diet K-Diet K (K-K) n = 3 females, n = 2 males. Diet C-Diet K (C-K) n = 3 females, n = 2 males. Diet K-Diet C (K-C) n = 3 females, n = 2 males. Diet C-Diet C (C-C) n = 4 females, n = 1 male.

In our study, pathways directly related to vitamin biosynthesis, metabolism, and transport, namely the COG “Coenzyme transport and metabolism” and KEGG Orthology (KO) “Metabolism of cofactors and vitamins” pathways, were significantly enriched (FDR < 0.05) in the metagenomes of kittens fed the canned diet (Table 4) compared to the kibbled diet. Within KO “Metabolism of cofactors and vitamins”, five pathways were differentially represented (FDR < 0.05) and of those, four were related to metabolism of vitamin B; “Thiamine metabolism” (PATH:ko00730), “Riboflavin metabolism” (PATH:ko00740), “Folate biosynthesis” (PATH:ko00790), and “Biotin metabolism” (PATH:ko00780; Fig. 4). Of these B-vitamin related pathways, all except “Folate biosynthesis” were more relatively abundant in kittens fed the canned diet.

The most relatively abundant vitamin B-related sequences included those with hits to ThiH (K03150), involved in thiamine biosynthesis, and RibB (EC:4.1.99.12), required for riboflavin biosynthesis (Table 5). Both *thiH* and *ribB* occurred in metagenomes with a prevalence between 0.26% and 0.70% in K-C and C-C kittens, but <0.12% in K-K and C-K kittens. Four other genes involved in riboflavin biosynthesis, predicted to encode UbiB (EC:1.16.1.3/EC:1.5.1.41), RibH (EC:2.5.1.78), SsuE (EC:1.5.1.38 ) and AphA (EC:3.1.3.2), were also more abundant in kittens fed the canned diet (FDR < 0.05; Table 5). Of these genes, *ubiB*, *ssuE* and *aphA* were also affected by the maternal diet, and occurred with the greatest relative abundance in K-C kittens (interaction FDR < 0.01). UbiB and SsuE are also involved in the conversion of riboflavin to other metabolites, which may explain the reduced faecal and urinary riboflavin concentrations observed when feeding low carbohydrate diets in cats^57^. In humans, *Bacteroidetes*, *Fusobacteria* and *Proteobacteria* are the dominant phyla responsible for riboflavin synthesis^58^ and these phyla collectively formed a significantly larger proportion of the microbiota in kittens fed the canned diet. In contrast, the genes with predicted involvement in folate biosynthesis, encoding FolC (EC:6.3.2.12) and SulD (FolB; EC:4.1.2.25), were relatively more abundant in kittens fed the kibbled diet compared to the canned diet (FDR < 0.05; Table 5). Both *folC*, which encodes dihydrofolate/folylpolyglutamate synthase, and *suID* which encodes dihydroneopterin aldolase, are prevalent among *Streptococcus* and *Lactobacillus* genomes^59^,^60^,^61^,^62^,^63^, and these taxa were significantly enriched in kittens fed the kibbled diet. The increased metagenome potential for synthesising thiamine and riboflavin in kittens fed the canned diet, but increased potential for folate synthesis in kibble-fed kittens, suggests scope for improving both types of diets to optimise microbial vitamin B biosynthesis in the cat.

In addition to changes in vitamin B-related functions, the post-weaning diet also affected proportions of vitamin K-related genes and functions in the faecal metagenomes (Table 5). Vitamin K, an essential vitamin for the cat, is fat soluble and is required for a number of functions including blood coagulation. Vitamin K comes in two natural forms - phylloquinone (vitamin K1) and menaquinone (vitamin K2). Phylloquinone, also known as the plant form of vitamin K, is converted to menaquinone in the intestine^64^. The KO pathway encompassing menaquinone biosynthesis and conversion is associated with the “Ubiquinone and other terpenoid-quinone biosynthesis” (PATH:ko00130) pathway, which was significantly enriched in kittens fed the canned diets (FDR < 0.001; Table 5). Within this pathway, five enzymes involved in menaquinone biosynthesis, MenA (EC:2.5.1.74), MenG (EC:4.1.3.36), MenD (EC:2.2.1.9), MenF (EC:5.4.4.2), and MenH (EC:4.2.99.20), showed significant differences (FDR < 0.05) in representation between kittens fed the canned and kibbled diet. Of these, all but *menA* were relatively more abundant in kittens fed the canned diet. These differences raise the possibility that more vitamin K2 is being produced by the microbial community in kittens fed the canned diet.

### Taurine metabolism

Taurine is an essential nutrient for the cat and therefore must be supplied by all complete and balanced diets. This is in part due to the high reliance the cat has on taurine to conjugate bile acids. The type of diet fed to the cat – i.e., kibbled or canned, determines the required level of taurine required to be supplied by the diet. For example, 1 g taurine per kg DM is required in kibbled diets whereas canned diets require 2 g taurine per kg DM diet^50^. Additionally, the relationship between taurine and Maillard reaction products and intestinal microbiota has been of interest for the cat since work published in 1996 showed that Maillard reaction products induced taurine depletion in the cat and that this could be reversed with antibiotics^65^.

Taurine is readily degraded by the intestinal microbiota^7^,^8^. The main enzyme responsible for taurine degradation is α-ketogluterate amino transferase (EC 2.6.1.55). In the current study, the levels of genes encoding two bacterial enzymes involved in taurine breakdown (taurine dioxygenase [EC 1.14.11.17; TauD] and sulfoacetaldehyde acetyltransferase [EC:2.3.3.15; Xsc]^66^,^67^,^68^) were increased in cats fed canned diets post-weaning, although both were still at low levels. This may support results from previous studies that suggest taurine may be broken down to sulphite at higher rates in canned diets^7^,^8^.

Cats preferentially use taurine to conjugate bile acids. The principal bile acid in cats is taurocholic acid, and the bacterial degradation of taurocholic acid leads to loss of taurine, thus increasing the dietary requirement. Previous work has shown that cats fed kibbled diets had a reduction in the levels and composition (primary and secondary bile salts) of faecal bile acid secretion compared to cats fed canned diets^7^. In the cat, secondary bile salts are produced as a result of 7-α-dehydroxylase activity^69^. Mining the KEGG pathways showed no detection of enzymes associated with bile salt biosynthesis such as cholyltaurine hydrolase (bilesalt dehydrogenase, choloylglycine hydrolase [EC:3.5.1.24]) or penicillin amidase [Pva; EC:3.5.1.11]-related sequences which may be annotated incorrectly as bile salt dehydrogenase in public databases^70^.

Recent reviews have shown that Maillard reaction products are high in commercially available pet foods, especially canned formulations^9^ and that they have the potential to impact on the health of the pet^10^. Maillard reaction products are thought to be degraded by a number of bacterial species, primarily through enzymes such as fructosamine 3-kinase (FN3K), fructosyl amino oxidase (FAP) and glucosamine-6-phospate synthase (GlmS). Only GlmS was identified in KEGG, and there was no effect of dietary treatment (FDR = 0.138). The relatively small differences associated with dietary format and taurine metabolism were surprising but may reflect that while analysing the metagenome provides information on the functional potential of the microbiome, differences in microbiome activity are not picked up by these analyses.

Given the changes observed in both vitamin B and K metabolism associated with dietary format, the impact of dietary format on the long term nutrition via vitamin status of the cat is one that should be investigated in future studies.

## Conclusion

To our knowledge this is the first time the effects of pre- and post-weaning diets on the genetic functional potential of the faecal microbiota have been investigated in the domestic kitten. Although the mother’s diet did not significantly alter the composition of the microbiome, a significant interaction with post-weaning diet was observed. The impact of post-weaning diet on the metagenome in terms of energy and carbohydrate metabolism, and vitamin biosynthesis functions, suggests that modulation of microbiome function through diet may be an important avenue for improving the nutrition of companion animals.

## Materials and Methods

### Ethics statement

All procedures undertaken on the cats were performed in accordance with relevant guidelines and regulations of the Animal Welfare (Companion Cats) Code of Welfare 2007 and were approved by the Massey University Animal Ethics Committee (MUAEC 10/108). All cats used were owned by Massey University and were housed at the Centre for Feline Nutrition (Massey University, Palmerston North, New Zealand) according to the Animal Welfare (Companion Cats) Code of Welfare 2007.

### Animals and housing

Animals and housing have been described in detail previously^6^. Briefly, queens were maintained on a moderate protein:fat:carbohydrate kibbled diet (35:20:28% DM; n = 4) or a high protein:fat:carbohydrate canned diet (45:37:2% DM; n = 3) throughout pregnancy and lactation – defined as the pre-weaning phase. From week 0 to 4, kittens received milk from their dam exclusively. At 4 weeks of age, the kittens were randomly assigned to one of the two diets (canned or kibble), and were weaned onto solid food in a gradual manner, receiving both their allocated diet and the dam’s milk until week 8, when the kittens were fully weaned. Half of each litter was randomly assigned (within sex) onto Diet C and half onto Diet K, forming four dietary treatment groups (C-C, C-K, K-C, K-K; n = 5 per treatment group; Table 6). The post-weaning phase was defined as commencing when the kitten was no longer receiving milk from its mother (8 weeks of age), and at this point kittens were removed from their mothers and placed into group housing according to their post-weaning diet. Diet and water were available *ad libitum* daily to allow for normal growth^6^.

### Diets

Commercially available kibbled (Diet K; moderate protein:fat:carbohydrate - 35:20:28% DM) and canned diets (Diet C; protein:fat:carbohydrate - 45:37:2% DM) were utilised in this study (Table 7). Both diets were formulated to meet the nutrient requirements for growth, gestation and lactation according to the Association of American Feed Control Officials. Diets were analysed for moisture content using a convection oven at 105 °C (AOAC 930.15, 925.10) and the ash residue using a furnace at 550 °C (AOAC 942.05). Crude protein and crude fat were determined using the Leco total combustion method (AOAC 968.06) and acid hydrolysis/Mojonnier extraction (AOAC 954.02), respectively. Gross energy (kJ/g) was determined using bomb calorimetry. Crude fibre was determined using the gravimetric method (AOAC 978.10) and Nitrogen-Free Extractable matter (NFE) by difference^6^.

### Digestibility

Apparent digestibility of dietary macronutrients (energy, protein, fat) was determined during week 17. Individual total food intake and refusals were recorded daily and total faecal output was collected over a five day period and frozen (−20 °C), freeze dried, and ground for analysis. The diet and faeces were analysed for moisture using a convection oven at 105 °C (AOAC 930.15, 925.10), ash using a furnace at 550 °C (AOAC 942.05). Crude protein and crude fat were determined using the Leco total combustion method (AOAC 968.06) and acid hydrolysis/Mojonnier extraction (AOAC 954.02), respectively. Gross energy (kJ/g) was determined using bomb calorimetry.

### DNA extraction

At 17 weeks of age, the kittens were housed individually for 24 h and a fresh faecal sample was collected from each animal within 15 min of excretion, snap-frozen in liquid nitrogen and stored at −85 °C^6^. High molecular weight metagenomic DNA was extracted from faecal samples using a non-mechanical lysis method as follows. One hundred mg of faecal material was incubated in 300 μl of a lysozyme and RNAse solution (40 mg lysozyme [Sigma L6876; Sigma-Aldrich, St. Louis, MO, United States] + 1 ml 10 mM Tris HCl/1 mM EDTA + 15 μl RNAse cocktail [Ambion AM2286; Thermo Fisher Scientific, Waltham, MA 02451, USA]) for 30 min at room temperature. Afterwards, 60 μl of Proteinase K solution (Ambion AM2546) was added to each tube and incubated for a further 30 min at room temperature. Extraction of DNA was completed using Nucleospin Soil kits (Macherey-Nagel, Düren, Germany) following the manufacturer’s instructions.

### Sequence analyses and statistics

Metagenomic DNA extracted from faeces (week 17) was analysed by paired-end (PE100) shotgun sequencing using the Illumina Hi-Seq 2000 platform from Macrogen Inc. (Seoul, Republic of Korea), a commercial sequencing provider. Paired-end sequences were joined *in silico*, and taxonomically and functionally classified using the MG-RAST pipeline (version 3.2)^71^. Taxonomic assignments were carried out using the best hit classification method with a minimum 80% identity cut off against the M5NR database^72^. Functional classifications were predicted from the COG and KO databases with a 0.8 identity threshold. COG and KO functions that did not occur in at least 5 samples with a relative abundance greater than 1 × 10^−8^ were removed from the dataset. Similarly, only taxa that occurred in at least 5 samples with a relative abundance greater than 1 × 10^−4^ were included.

Statistical tests were performed using R 3.1.3^73^. Difference between mean relative abundances of taxa and predicted functions were analysed by two-factor permutation ANOVA implemented using the RVAideMemoire package (version 0.9-45-2)^74^. Adjustment of *P* values for multiple testing was performed using the Benjamini & Hochberg false discovery rate (FDR) method, with FDR < 0.05 considered significant. Permutation MANOVA and Procrustes rotation analysis was performed using the adonis and procrustes functions, respectively, as implemented in the vegan package^75^. Partial least squares discriminant analysis (PLS-DA) and principal component analysis was performed using the mixOmics package^77^.

## Additional Information

**How to cite this article**: Young, W. *et al*. Pre- and post-weaning diet alters the faecal metagenome in the cat with differences in vitamin and carbohydrate metabolism gene abundances. *Sci. Rep.* **6**, 34668; doi: 10.1038/srep34668 (2016).

**Publisher’s note:** Springer Nature remains neutral with regard to jurisdictional claims in published maps and institutional affiliations.

## Supplementary Material

Supplementary Information

## Figures and Tables

**Table 1:** The effects of pre-weaning and post-weaning diets on the energy, fat and protein intake and content in faeces and apparent digestibility of energy, fat and protein in the domestic kitten (n = 5 per treatment).

	K-K		K-C		C-K		C-C		P-value		
Macronutrient	Mean	SEM	Mean	SEM	Mean	SEM	Mean	SEM	Pre^1^	Post^2^	Pre*post
Energy											
Apparent digestibility	74.0	4.7	82.5	1.7	73.7	3	80.5	1.5	0.583	0.002	0.682
Energy intake (kJ)	0.36	0.03	0.82	0.1	0.48	0.03	1.23	0.12	0.004	<0.001	0.86
Content of faeces (kJ)	0.09	0.02	0.15	0.03	0.13	0.022	0.26	0.042	0.019	0.006	0.255
Fat											
Apparent digestibility	80.4	2.1	90.6	1.8	80.1	4.3	82.5	3.7	0.203	0.063	0.239
Fat intake (g DM/d)	9.5	0.8	32.9	4	12.7	0.7	49.4	4.7	0.006	<0.001	0.049
Content of faeces (g DM/d)	1.8	0.09	3.3	1.3	2.62	0.7	9.22	2.6	0.032	0.012	0.096
Protein											
Apparent digestibility	71.7	1.6	89	0.6	70.8	3.2	86.9	0.8	0.428	0.001	0.772
Protein intake (g DM/d)	16.6	1.4	39.7	4.8	22.2	1.3	59.7	5.7	0.004	<0.001	0.08
Content of faeces (g DM)	4.62	0.21	4.44	0.76	6.56	0.99	7.92	1.17	0.006	0.503	0.385

Data are presented as the mean and standard error of the mean (SEM). P value indicates ANOVA significance of rank transformed data.

K = Kibbled.

C = Canned.

Diet K-Diet K (K-K) n = 3 females, n = 2 males.

Diet C-Diet K (C-K) n = 3 females, n = 2 males.

Diet K-Diet C (K-C) n = 3 females, n = 2 males.

Diet C-Diet C (C-C) n = 4 females, n = 1 male.

^1^Comparisons between Diets K-K and K-C vs C-K and C-C.

^2^Comparison between Diets K-K and C-K and C-C and K-C.

DM = dry matter.

**Table 2:** The effects of pre-weaning or post-weaning diets (canned or kibbled) on the bacterial genera (proportion of total sequences) present in faecal samples of the domestic kitten *(Felis catus*; n = 5 per treatment).

	K-K		K-C		C-K		C-C		Pre-weaning diet^1^		Post-weaning diet^2^		Pre x Post-weaning	
Genus	Mean	SEM	Mean	SEM	Mean	SEM	Mean	SEM	P-value	FDR	P-value	FDR	P-value	FDR
*Bifidobacterium*	6.07	3.45	0.05	0.01	22.81	9.39	0.04	0.00	0.094	0.376	0.001	0.004	0.085	0.315
*Bacteroides*	0.15	0.04	25.54	8.42	1.98	1.14	38.31	9.33	0.272	0.456	0.001	0.004	0.363	0.571
*Parabacteroides*	0.01	0.00	0.91	0.34	0.09	0.06	1.18	0.29	0.417	0.602	0.001	0.004	0.684	0.847
*Alistipes*	0.00	0.00	0.11	0.04	0.02	0.02	0.22	0.08	0.165	0.414	0.001	0.004	0.337	0.565
*Lactobacillus*	10.63	4.22	0.03	0.01	22.34	8.72	0.02	0.00	0.270	0.456	0.001	0.004	0.263	0.489
*Clostridium*	1.42	0.42	7.50	1.79	2.07	0.41	13.84	9.16	0.727	0.860	0.001	0.004	0.863	0.984
uncl *Clostridiales*	0.20	0.02	1.01	0.20	0.30	0.05	0.64	0.07	0.231	0.414	0.001	0.004	0.046	0.199
*Phascolarctobacterium*	0.01	0.01	0.84	0.13	0.16	0.14	0.99	0.09	0.201	0.414	0.001	0.004	0.976	0.984
*Fusobacterium*	0.08	0.07	4.87	1.95	0.04	0.04	4.75	0.99	0.955	0.972	0.001	0.004	0.973	0.984
*Citrobacter*	0.00	0.00	0.06	0.02	0.00	0.00	0.01	0.01	0.005	0.043	0.001	0.004	0.010	0.087
*Enterobacter*	0.00	0.00	0.04	0.02	0.00	0.00	0.01	0.00	0.006	0.045	0.001	0.004	0.003	0.031
*Salmonella*	0.01	0.01	0.18	0.07	0.02	0.01	0.02	0.00	0.010	0.065	0.001	0.004	0.002	0.026
*Shigella*	0.08	0.04	3.15	1.21	0.10	0.08	0.11	0.08	0.001	0.017	0.001	0.004	0.001	0.026
*Finegoldia*	0.08	0.04	0.00	0.00	0.01	0.00	0.00	0.00	0.055	0.286	0.002	0.005	0.034	0.189
*Faecalibacterium*	0.16	0.08	1.78	0.42	0.30	0.14	1.28	0.35	0.539	0.684	0.002	0.005	0.273	0.489
*Campylobacter*	0.03	0.01	0.16	0.04	0.06	0.02	0.13	0.03	0.823	0.951	0.002	0.005	0.260	0.489
unclassified	0.01	0.00	0.06	0.01	0.04	0.01	0.06	0.01	0.114	0.395	0.002	0.005	0.113	0.377
*Gardnerella*	0.02	0.01	0.00	0.00	0.04	0.01	0.00	0.00	0.143	0.414	0.002	0.005	0.170	0.421
*Collinsella*	10.01	4.49	0.63	0.21	12.93	2.37	1.81	1.43	0.449	0.630	0.003	0.006	0.731	0.864
*Streptococcus*	46.52	18.26	0.25	0.07	0.13	0.02	0.10	0.01	0.001	0.017	0.003	0.006	0.002	0.026
uncl *Ruminococcaceae*	0.07	0.03	0.56	0.05	0.19	0.13	0.33	0.06	0.463	0.633	0.003	0.006	0.039	0.189
*Escherichia*	0.12	0.06	4.48	1.93	0.18	0.14	0.17	0.11	0.001	0.017	0.003	0.006	0.001	0.026
*Kineococcus*	0.00	0.00	0.00	0.00	0.01	0.00	0.00	0.00	0.090	0.376	0.003	0.006	0.116	0.377
*Desulfovibrio*	0.00	0.00	0.39	0.22	0.04	0.02	0.39	0.17	0.874	0.967	0.005	0.010	0.900	0.984
*Coprobacillus*	0.02	0.01	0.19	0.07	0.04	0.01	0.05	0.02	0.085	0.376	0.007	0.014	0.025	0.162
*Holdemania*	0.00	0.00	0.37	0.19	0.03	0.03	0.11	0.05	0.313	0.496	0.008	0.015	0.165	0.421
*Megamonas*	0.01	0.01	1.10	0.42	0.24	0.13	0.93	0.45	0.934	0.971	0.011	0.020	0.543	0.721
*uncl Siphoviridae*	0.05	0.01	0.00	0.00	0.01	0.00	0.01	0.01	0.227	0.414	0.012	0.021	0.040	0.189
*Dehalococcoides*	0.00	0.00	0.04	0.01	0.02	0.01	0.02	0.01	0.843	0.953	0.017	0.028	0.060	0.240
*Megasphaera*	0.91	0.77	0.01	0.01	0.90	0.40	0.00	0.00	0.924	0.971	0.025	0.041	0.939	0.984
*Klebsiella*	0.03	0.02	0.05	0.02	0.00	0.00	0.00	0.00	0.003	0.039	0.353	0.426	0.416	0.584

Results are presented as mean and standard error of the mean (SEM). P value indicates ANOVA significance of rank transformed data and False Discovery Rate (FDR) indicates multiple testing adjusted P value. Taxa shown are those with significant differences in relative abundance between post-weaning treatments with the highest mean relative abundances across all groups.

K = Kibbled.

C = Canned.

Diet K-Diet K (K-K) n = 3 females, n = 2 males.

Diet C-Diet K (C-K) n = 3 females, n = 2 males.

Diet K-Diet C (K-C) n = 3 females, n = 2 males.

Diet C-Diet C (C-C) n = 4 females, n = 1 male.

^1^Comparisons between Diets K-K and K-C vs C-K and C-C.

^2^Comparison between Diets K-K and C-K and C-C and K-C.

**Table 3:** The effects of pre-weaning (gestation and lactation) or post-weaning diets (canned or kibbled) on the KEGG orthology pathways (proportion of total sequences) present in faecal samples of the domestic kitten (*Felis catus*; n = 5 per treatment).

KEGG Orthology	K-K		K-C		C-K		C-C		Pre-weaning diet^1^		Post-weaning diet^2^		Pre x Post-weaning	
L1 L2	Mean	SEM	Mean	SEM	Mean	SEM	Mean	SEM	P-value	FDR	P-value	FDR	P-value	FDR
Cellular processes and signaling														
Intracellular trafficking etc	1.39	0.25	4.22	0.48	1.39	0.15	3.02	0.40	0.097	0.318	0.001	0.006	0.073	0.250
Posttranslational modification etc	5.52	0.60	3.88	0.27	4.07	0.25	3.45	0.12	0.007	0.084	0.002	0.010	0.185	0.403
Extracellular structures	0.00	0.00	0.05	0.02	0.00	0.00	0.00	0.00	0.005	0.084	0.003	0.012	0.001	0.024
Cell motility	0.04	0.02	0.36	0.18	0.01	0.01	0.13	0.10	0.256	0.559	0.010	0.030	0.416	0.623
Defense mechanisms	0.66	0.18	1.83	0.18	1.65	0.62	2.46	0.10	0.039	0.197	0.015	0.040	0.602	0.761
Signal transduction mechanisms	1.62	0.35	4.11	0.63	2.53	0.11	2.03	0.67	0.273	0.559	0.067	0.124	0.011	0.132
Cell cycle control, etc	1.41	0.21	0.79	0.17	1.18	0.19	1.16	0.15	0.745	0.908	0.106	0.169	0.132	0.343
Cell wall/membrane/envelope biogenesis	4.00	0.21	4.87	0.54	4.91	0.83	4.54	0.53	0.651	0.868	0.675	0.796	0.300	0.480
Cytoskeleton	0.00	0.00	0.00	0.00	0.00	0.00	0.00	0.00	0.165	0.440	0.701	0.796	0.256	0.469
Information storage and processing														
Replication, recombination and repair	9.06	0.59	5.78	0.64	8.95	0.22	7.87	0.28	0.041	0.197	0.001	0.006	0.023	0.184
RNA processing and modification	0.02	0.01	0.02	0.01	0.04	0.01	0.00	0.00	0.959	0.959	0.027	0.059	0.037	0.192
Translation, ribosomal structure etc	17.48	1.08	10.87	1.25	15.05	2.48	14.23	1.45	0.774	0.908	0.047	0.094	0.100	0.300
Transcription	5.23	0.27	5.59	0.80	5.48	0.44	4.23	0.34	0.311	0.574	0.361	0.474	0.143	0.343
Chromatin structure and dynamic	0.00	0.00	0.02	0.02	0.00	0.00	0.01	0.01	0.921	0.959	0.376	0.474	0.832	0.993
Coenzyme transport and metabolism	2.01	0.12	3.16	0.24	2.47	0.21	3.64	0.16	0.027	0.197	0.001	0.006	0.937	0.993
Energy production and conversion	4.94	0.20	10.48	0.47	4.49	0.40	9.87	0.97	0.380	0.595	0.001	0.006	0.902	0.993
Carbohydrate transport and metabolism	9.57	0.71	7.41	0.83	11.57	0.75	8.43	0.98	0.106	0.318	0.005	0.017	0.555	0.761
Nucleotide transport and metabolism	7.99	0.52	5.32	0.52	7.12	1.04	5.94	0.36	0.859	0.937	0.018	0.043	0.274	0.469
Inorganic ion transport and metabolism	3.19	0.34	4.96	0.39	3.78	0.69	3.59	0.18	0.397	0.595	0.097	0.166	0.047	0.192
Amino acid transport and metabolism	10.09	1.93	8.30	0.56	7.81	0.89	8.81	0.73	0.460	0.649	0.729	0.796	0.266	0.469
Secondary metabolites biosynthesis, etc	0.73	0.10	0.70	0.11	0.51	0.14	0.49	0.05	0.053	0.212	0.794	0.829	0.993	0.993
Lipid transport and metabolism	2.68	0.65	2.66	0.17	3.15	0.38	3.15	0.35	0.280	0.559	0.980	0.980	0.977	0.993
Poorly characterized														
General function prediction only	7.70	0.99	9.46	0.36	8.54	1.55	9.18	0.80	0.794	0.908	0.277	0.415	0.593	0.761
Function unknown	4.66	0.30	5.16	0.58	5.27	0.32	3.76	0.58	0.389	0.595	0.294	0.415	0.048	0.192

Results are presented as mean and standard error of the mean (SEM). *P value* indicates ANOVA significance of rank transformed data and False Discovery Rate (*FDR)* indicates multiple testing adjusted *P* value.

K = Kibbled.

C = Canned.

Diet K-Diet K (K-K) n = 3 females, n = 2 males.

Diet C- Diet K (C-K) n = 3 females, n = 2 males.

Diet K- Diet C (K-C) n = 3 females, n = 2 males.

Diet C- Diet C (C-C) n = 4 females, n = 1 male.

1. Comparisons between Diets K-K and K-C vs C-K and C-C.

2. Comparison between Diets K-K and C-K and C-C and K-C.

**Table 4:** The effects of pre-weaning (gestation and lactation) or post-weaning diets (canned or kibbled) on the Metabolism of cofactors and vitamins KEGG orthology pathway (proportion of total sequences) present in faecal samples of the domestic kitten (*Felis catus*; n = 5 per treatment).

KEGG Orthlology Level Three function	K-K		K-C		C-K		C-C		Pre-weaning diet^1^		Post-weaning diet^2^		Pre x Post-weaning	
Metabolism of cofactors and vitamins	Mean	SEM	Mean	SEM	Mean	SEM	Mean	SEM	P-value	FDR	P-value	FDR	P-value	FDR
Thiamine metabolism [PATH:ko00730]	0.51	0.08	1.31	0.15	0.55	0.13	1.20	0.12	0.786	0.968	0.001	0.006	0.541	0.650
Ubiquinone/other biosynthesis [PATH:ko00130]	0.09	0.03	0.35	0.05	0.14	0.05	0.28	0.05	0.813	0.968	0.001	0.006	0.204	0.586
Riboflavin metabolism [PATH:ko00740]	0.25	0.08	0.47	0.05	0.21	0.08	0.52	0.04	0.846	0.968	0.004	0.012	0.515	0.650
Folate biosynthesis [PATH:ko00790]	0.30	0.06	0.02	0.01	0.23	0.09	0.08	0.07	0.887	0.968	0.004	0.012	0.270	0.586
Biotin metabolism [PATH:ko00780]	0.06	0.02	0.16	0.05	0.06	0.03	0.14	0.04	0.742	0.968	0.017	0.041	0.817	0.817
Lipoic acid metabolism [PATH:ko00785]	0.10	0.03	0.02	0.01	0.04	0.02	0.04	0.01	0.252	0.968	0.054	0.108	0.074	0.586
One carbon pool by folate [PATH:ko00670]	0.49	0.10	0.69	0.12	0.67	0.08	0.80	0.08	0.163	0.968	0.110	0.172	0.722	0.788
Pantothenate/CoA biosynthesis [PATH:ko00770]	0.15	0.05	0.33	0.04	0.24	0.10	0.28	0.04	0.765	0.968	0.115	0.172	0.288	0.586
Vitamin B6 metabolism [PATH:ko00750]	0.21	0.05	0.32	0.03	0.31	0.05	0.32	0.06	0.382	0.968	0.216	0.275	0.324	0.586
Nicotinate/nicotinamide metabolism [PATH:ko00760]	0.77	0.18	0.43	0.10	0.70	0.23	0.61	0.14	0.763	0.968	0.229	0.275	0.470	0.650
Porphyrin/chlorophyll metabolism [PATH:ko00860]	0.20	0.08	0.20	0.06	0.05	0.02	0.49	0.36	0.974	0.974	0.257	0.280	0.236	0.586
Retinol metabolism [PATH:ko00830]	0.00	0.00	0.00	0.00	0.00	0.00	0.00	0.00	0.125	0.968	0.749	0.749	0.342	0.586

Results are presented as mean and standard error of the mean (SEM). *P value* indicates ANOVA significance of rank transformed data and False Discovery Rate (*FDR)* indicates multiple testing adjusted *P* value.

K = Kibbled.

C = Canned.

Diet K-Diet K (K-K) n = 3 females, n = 2 males.

Diet C-Diet K (C-K) n = 3 females, n = 2 males.

Diet K-Diet C (K-C) n = 3 females, n = 2 males.

Diet C-Diet C (C-C) n = 4 females, n = 1 male.

^1^Comparisons between Diets K-K and K-C vs C-K and C-C.

^2^Comparison between Diets K-K and C-K and C-C and K-C.

**Table 5:** The effects of pre-weaning (gestation and lactation) or post-weaning diets (canned or kibbled) on the enzymes involved in key vitamin biosynthesis and metabolism related pathways the Ubiquinone and other terpenoid-quinone biosynthesis [PATH:ko00130], Thiamine metabolism [PATH:ko00730], Sulfur relay system [PATH:ko04122], Riboflavin metabolism [PATH:ko00740], Vitamin B6 metabolism [PATH:ko00750], Nicotinate and nicotinamide metabolism [PATH:ko00760], Pantothenate and CoA biosynthesis [PATH:ko00770], Biotin metabolism [PATH:ko00780], and Folate biosynthesis [PATH:ko00790] KEGG orthology pathways (proportion of total sequences) present in faecal samples of the domestic kitten (*Felis catus*; n = 5 per treatment).

		K-K		K-C		C-K		C-C		Pre-weaning diet^1^		Post-weaning diet^2^		Pre x Post-weaning	
	KEGG ID	Mean	SEM	Mean	SEM	Mean	SEM	Mean	SEM	P-value	FDR	P-value	FDR	P-value	FDR
Ubiquinone and other terpenoid-quinone biosynthesis [PATH:ko00130]															
Menb; [EC:4.1.3.36]	K01661	0.003	0.001	0.136	0.033	0.055	0.029	0.168	0.036	0.143	0.321	0.003	0.008	0.726	0.838
Menh; [EC:4.2.99.20]	K08680	0.000	0.000	0.005	0.002	0.000	0.000	0.000	0.000	0.008	0.029	0.001	0.005	0.025	0.075
Ubib, aarf	K03688	0.086	0.035	0.008	0.008	0.046	0.046	0.033	0.027	0.820	0.914	0.214	0.269	0.267	0.466
Ubif; [EC:1.14.13.-]	K03184	0.000	0.000	0.012	0.005	0.000	0.000	0.000	0.000	0.001	0.007	0.001	0.005	0.001	0.005
Ubih; [EC:1.14.13.-]	K03185	0.000	0.000	0.009	0.003	0.001	0.000	0.000	0.000	0.001	0.007	0.001	0.005	0.001	0.005
Ubix; [EC:4.1.1.-]	K03186	0.000	0.000	0.007	0.004	0.000	0.000	0.000	0.000	0.009	0.032	0.048	0.076	0.041	0.111
Mend; [EC:2.2.1.9]	K02551	0.000	0.000	0.075	0.029	0.000	0.000	0.057	0.036	0.639	0.810	0.017	0.033	0.670	0.802
Menf; [EC:5.4.4.2]	K02552	0.000	0.000	0.009	0.004	0.000	0.000	0.001	0.001	0.003	0.013	0.001	0.005	0.007	0.024
Mena; [EC:2.5.1.74/EC:2.5.1.-]	K02548	0.000	0.000	0.000	0.000	0.033	0.012	0.000	0.000	0.003	0.013	0.002	0.006	0.003	0.012
Ubia; [EC:2.5.1.-]	K03179	0.000	0.000	0.008	0.004	0.001	0.001	0.000	0.000	0.027	0.083	0.068	0.100	0.012	0.039
Ubic; [EC:4.1.3.40]	K03181	0.000	0.000	0.007	0.002	0.000	0.000	0.000	0.000	0.001	0.007	0.001	0.005	0.001	0.005
Ubie; [EC:2.1.1.163/EC:2.1.1.201]	K03183	0.000	0.000	0.023	0.010	0.004	0.002	0.016	0.016	0.891	0.955	0.097	0.134	0.591	0.753
Entc; [EC:5.4.4.2]	K02361	0.025	0.015	0.011	0.007	0.000	0.000	0.000	0.000	0.019	0.061	0.322	0.378	0.342	0.538
Ubig; [EC:2.1.1.222/EC:2.1.1.64]	K00568	0.000	0.000	0.005	0.002	0.000	0.000	0.000	0.000	0.001	0.007	0.008	0.018	0.003	0.012
Ubid; [EC:4.1.1.-]	K03182	0.001	0.001	0.024	0.013	0.000	0.000	0.000	0.000	0.007	0.025	0.034	0.057	0.029	0.084
Thiamine metabolism [PATH:ko00730]															
Thil; [EC:2.7.4.16]	K01516	0.090	0.037	0.144	0.051	0.093	0.036	0.191	0.025	0.529	0.730	0.063	0.094	0.569	0.742
Thi80; [ec:2.7.6.2]	K00949	0.036	0.015	0.000	0.000	0.010	0.010	0.007	0.007	0.317	0.532	0.043	0.069	0.105	0.239
Tena; [EC:3.5.99.2]	K03707	0.001	0.000	0.003	0.003	0.034	0.015	0.009	0.009	0.052	0.145	0.241	0.296	0.112	0.250
Thig	K03149	0.135	0.076	0.327	0.051	0.197	0.056	0.264	0.043	0.994	0.996	0.049	0.077	0.303	0.501
Thih	K03150	0.105	0.096	0.703	0.106	0.117	0.080	0.530	0.056	0.367	0.573	0.001	0.005	0.307	0.503
Thik; [EC:2.7.1.89]	K07251	0.000	0.000	0.004	0.001	0.000	0.000	0.000	0.000	0.001	0.007	0.001	0.005	0.001	0.005
Sufs; [EC:2.8.1.7/EC:4.4.1.16]	K11717	0.142	0.058	0.128	0.059	0.029	0.029	0.164	0.068	0.539	0.737	0.297	0.354	0.181	0.360
Sulfur relay system [PATH:ko04122]															
Thif; [EC:2.7.7.73]	K03148	0.012	0.012	0.004	0.004	0.031	0.017	0.014	0.014	0.269	0.493	0.354	0.411	0.652	0.788
Thii	K03151	0.101	0.041	0.007	0.007	0.041	0.029	0.022	0.022	0.374	0.579	0.049	0.077	0.229	0.420
This	K03154	0.003	0.003	0.000	0.000	0.009	0.004	0.004	0.004	0.155	0.341	0.216	0.271	0.740	0.848
Riboflavin metabolism [PATH:ko00740]															
Fre, ubib; [EC:1.16.1.3/EC:1.5.1.41]	K05368	0.000	0.000	0.009	0.003	0.000	0.000	0.000	0.000	0.001	0.007	0.001	0.005	0.001	0.005
Ribb, RIB3; [EC:4.1.99.12]	K02858	0.085	0.030	0.265	0.024	0.099	0.069	0.307	0.041	0.559	0.751	0.002	0.006	0.745	0.851
Ribd; [EC:3.5.4.26/EC:1.1.1.193]	K11752	0.070	0.029	0.000	0.000	0.025	0.015	0.020	0.020	0.501	0.705	0.058	0.088	0.100	0.232
Ribe, RIB5; [EC:2.5.1.9]	K00793	0.042	0.017	0.065	0.029	0.000	0.000	0.073	0.020	0.427	0.631	0.034	0.057	0.194	0.377
Ribh, RIB4; [EC:2.5.1.78]	K00794	0.050	0.010	0.115	0.011	0.041	0.023	0.124	0.019	0.977	0.986	0.001	0.005	0.607	0.761
Ssue; [EC:1.5.1.38]	K00299	0.000	0.000	0.004	0.001	0.000	0.000	0.000	0.000	0.001	0.007	0.002	0.006	0.002	0.008
Apha; [EC:3.1.3.2]	K03788	0.000	0.000	0.008	0.003	0.000	0.000	0.000	0.000	0.006	0.022	0.002	0.006	0.002	0.008
Epd; [EC:1.2.1.72]	K03472	0.000	0.000	0.013	0.005	0.000	0.000	0.000	0.000	0.004	0.017	0.001	0.005	0.002	0.008
Pdxs, pdx1; [EC:4.-.-.-]	K06215	0.210	0.054	0.207	0.029	0.252	0.060	0.157	0.025	0.922	0.968	0.261	0.317	0.297	0.496
Pdxj; [EC:2.6.99.2]	K03474	0.000	0.000	0.055	0.010	0.001	0.001	0.066	0.018	0.569	0.759	0.001	0.005	0.601	0.758
Nicotinate and nicotinamide metabolism [PATH:ko00760]															
Iunh; [EC:3.2.2.1]	K01239	0.000	0.000	0.002	0.002	0.118	0.052	0.017	0.017	0.007	0.025	0.107	0.145	0.087	0.208
Nada; [EC:2.5.1.72]	K03517	0.065	0.027	0.077	0.052	0.000	0.000	0.117	0.059	0.773	0.885	0.145	0.190	0.224	0.417
Nadc, QPRT; [EC:2.4.2.19]	K00767	0.066	0.027	0.175	0.027	0.000	0.000	0.190	0.026	0.283	0.509	0.001	0.005	0.087	0.208
Nadd; [EC:2.7.7.18]	K00969	0.074	0.036	0.000	0.000	0.030	0.018	0.009	0.009	0.438	0.643	0.015	0.030	0.224	0.417
Pncb, NAPRT1; [EC:2.4.2.11]	K00763	0.165	0.067	0.000	0.000	0.150	0.065	0.027	0.027	0.901	0.960	0.013	0.026	0.640	0.788
Pnta; [EC:1.6.1.2]	K00324	0.023	0.013	0.016	0.003	0.060	0.021	0.003	0.002	0.342	0.553	0.016	0.031	0.052	0.136
Pntb; [EC:1.6.1.2]	K00325	0.037	0.022	0.013	0.003	0.089	0.017	0.007	0.002	0.137	0.311	0.004	0.010	0.054	0.140
Ppnk, NADK;[EC:2.7.1.23]	K00858	0.060	0.025	0.014	0.014	0.064	0.039	0.012	0.012	0.982	0.988	0.071	0.103	0.928	0.968
Puna; [EC:2.4.2.1]	K03783	0.061	0.025	0.083	0.038	0.000	0.000	0.126	0.033	0.723	0.858	0.019	0.036	0.070	0.174
Stha, udha; [EC:1.6.1.1]	K00322	0.000	0.000	0.014	0.005	0.000	0.000	0.000	0.000	0.002	0.010	0.002	0.006	0.001	0.005
Yjjg; [EC:3.1.3.5]	K08723	0.000	0.000	0.007	0.003	0.000	0.000	0.000	0.000	0.001	0.007	0.001	0.005	0.001	0.005
5’-nucleotidase; [EC:3.1.3.5]	K01081	0.001	0.000	0.007	0.007	0.008	0.005	0.000	0.000	0.811	0.908	0.767	0.807	0.220	0.411
Nade; [EC:6.3.1.5]	K01916	0.152	0.014	0.000	0.000	0.068	0.037	0.000	0.000	0.054	0.150	0.001	0.005	0.048	0.128
Pnca; [EC:3.5.1.19 3.5.1.-]	K08281	0.011	0.007	0.000	0.000	0.062	0.029	0.000	0.000	0.086	0.222	0.001	0.005	0.075	0.184
Usha; [EC:3.1.3.5 3.6.1.45]	K11751	0.000	0.000	0.013	0.007	0.000	0.000	0.000	0.000	0.017	0.055	0.039	0.063	0.013	0.042
Pantothenate and CoA biosynthesis [PATH:ko00770]															
Acps; [EC:2.7.8.7]	K00997	0.027	0.011	0.003	0.003	0.024	0.015	0.008	0.008	0.863	0.939	0.063	0.094	0.662	0.795
Pand; [EC:4.1.1.11]	K01579	0.000	0.000	0.062	0.006	0.010	0.008	0.057	0.012	0.704	0.852	0.001	0.005	0.370	0.569
Pane, apba; [EC:1.1.1.169]	K00077	0.080	0.023	0.016	0.005	0.026	0.019	0.016	0.011	0.100	0.245	0.043	0.069	0.125	0.275
Coaa; [EC:2.7.1.33]	K00867	0.045	0.018	0.014	0.006	0.057	0.030	0.000	0.000	0.958	0.982	0.031	0.054	0.464	0.654
Coaw; [EC:2.7.1.33]	K09680	0.000	0.000	0.009	0.004	0.000	0.000	0.002	0.002	0.115	0.270	0.019	0.036	0.088	0.209
Panb; [EC:2.1.2.11]	K00606	0.000	0.000	0.080	0.020	0.004	0.004	0.085	0.025	0.751	0.876	0.001	0.005	0.989	0.995
Panc; [EC:6.3.2.1]	K01918	0.000	0.000	0.114	0.005	0.000	0.000	0.068	0.028	0.158	0.344	0.001	0.005	0.160	0.326
Acph; [EC:3.1.4.14]	K08682	0.000	0.000	0.006	0.002	0.000	0.000	0.000	0.000	0.002	0.010	0.004	0.010	0.003	0.012
Biotin metabolism [PATH:ko00780]															
Biod; [EC:6.3.3.3]	K01935	0.000	0.000	0.025	0.011	0.000	0.000	0.008	0.008	0.332	0.544	0.031	0.054	0.346	0.542
Bira; [EC:6.3.4.15]	K03524	0.059	0.024	0.000	0.000	0.018	0.018	0.014	0.014	0.323	0.537	0.051	0.079	0.118	0.262
Biof; [EC:2.3.1.47]	K00652	0.002	0.001	0.112	0.032	0.041	0.033	0.093	0.038	0.689	0.842	0.019	0.036	0.307	0.503
Folate biosynthesis [PATH:ko00790]															
Folc; [EC:6.3.2.12 6.3.2.17]	K11754	0.147	0.060	0.000	0.000	0.060	0.037	0.023	0.023	0.418	0.622	0.023	0.042	0.174	0.348
Folp; [EC:2.5.1.15]	K00796	0.054	0.022	0.000	0.000	0.020	0.017	0.010	0.010	0.410	0.613	0.045	0.072	0.144	0.302
Paba; [EC:2.6.1.85]	K01664	0.029	0.017	0.008	0.004	0.043	0.016	0.009	0.007	0.526	0.727	0.037	0.061	0.592	0.753
Pabc; [EC:4.1.3.38]	K02619	0.000	0.000	0.003	0.003	0.034	0.017	0.009	0.009	0.038	0.111	0.275	0.331	0.198	0.382
Suld; [EC:4.1.2.25/EC:2.7.6.3]	K13940	0.038	0.024	0.000	0.000	0.070	0.024	0.008	0.008	0.287	0.511	0.020	0.037	0.474	0.660

Results are presented as mean and standard error of the mean (SEM). *P value* indicates ANOVA significance of rank transformed data and False Discovery Rate (*FDR)* indicates multiple testing adjusted *P* value.

K = Kibbled.

C = Canned.

Diet K-Diet K (K-K) n = 3 females, n = 2 males.

Diet C-Diet K (C-K) n = 3 females, n = 2 males.

Diet K-Diet C (K-C) n = 3 females, n = 2 males.

Diet C-Diet C (C-C) n = 4 females, n = 1 male.

**Table 6:** Treatment groups for determining the effects of pre-weaning (*in utero* and during lactation) and post-weaning diet on intestinal microbiota in the domestic kitten (*Felis catus*; n = 5 per treatment).

Queen diet		Post-weaning diet	
		Diet K	Diet C
Pre-weaning diet	Diet K	K-Kn = 3 females, n = 2 males	C-Kn = 3 females, n = 2 males
	Diet C	K-Cn = 3 females, n = 2 males	C-Cn = 4 females, n = 1 male

**Table 7:** Macronutrient profile of the kibbled (Diet K) and canned (diet C) diets fed to domestic short hair kittens (*Felis catus*)^1^.

Component	Diet K^2^	Diet C^3^
Dry Matter (DM; % as is)	92.9	31.7
Crude Protein (% DM)	35.3	45.3
Crude Fat (% DM)	20.2	37.6
Ash (% DM)	7.5	7.2
Crude Fibre (% DM)	1.8	1.5
NFE^4^ (% DM)	28.2	2.0
Gross energy (kcal/100 g DM)	507.0	618.1
Metabolisable energy^5^ (kcal/100 g DM)	393.8	485.1

^1^Both diets were formulated to meet the nutrient requirements for growth, gestation and lactation according to the Association of American Feed Control Officials (AAFCO).

^2^Ingredient list of Diet K (from pack): Corn, chicken and chicken meal, chicken digest, maize gluten, chicken tallow, tuna meal, poultry and poultry meal, iodinised salt, vegetable oil.

^3^Ingredient list of Diet C (from pack): Meat by-products and meat derived from chicken, lamb, beef, and mutton; gelling agent; minerals; vegetable oil, emulsifier; colouring; vitamins, chelating agents.

^4^Nitrogen free extract (% DM) calculated by difference (100 - crude protein - crude fat - crude fibre - ash).

^5^Determined using modified Atwater factors of: crude protein (3·5 kcal ME/g DM), crude fat (8·5 kcal ME/g DM), NFE (3·5 kcal ME/g DM).
