# Corrigendum: Pre- and post-weaning diet alters the faecal metagenome in the cat with differences in vitamin and carbohydrate metabolism gene abundances

**DOI:** 10.1038/srep41198

**Published:** 2017-01-24

**Authors:** Wayne Young, Christina D. Moon, David G. Thomas, Nick J. Cave, Emma N. Bermingham

Scientific Reports 6: Article number: 3466810.1038/srep34668; published online: 11 23 2016; updated: 01 24 2017

The original version of this Article contained a typographical error in the title, where:

“Pre- and post-weaning diet alters the faecal metagenome in the cat with differences vitamin and carbohydrate metabolism gene abundances”.

now reads:

“Pre- and post-weaning diet alters the faecal metagenome in the cat with differences in vitamin and carbohydrate metabolism gene abundances”.

